# Effect of Different Kinesio Taping Interventions on the Local Thresholds of Current Perception and Pressure Pain in Healthy Adults

**DOI:** 10.3389/fphys.2020.596159

**Published:** 2020-11-12

**Authors:** Kun Liu, Lulu Yin, Zheng Ma, Bo Yu, Yanhong Ma, Lihua Huang

**Affiliations:** ^1^Department of Rehabilitation Medicine, Shanghai Jiao Tong University Affiliated Sixth People’s Hospital, Shanghai, China; ^2^Department of Critical Care Medicine, Shanghai Tenth People’s Hospital, School of Medicine, Tongji University, Shanghai, China; ^3^Department of Rehabilitation, School of International Medical Technology, Sanda University, Shanghai, China

**Keywords:** Kinesio taping, taping methods, current perception threshold, pressure pain threshold, erector spinae

## Abstract

**Objective:**

Previous studies made controversial claims about the alleged effects of Kinesio taping (KT) on pain relief. To date, the mechanism by which KT relieves pain remains unclear. Moreover, pain evaluation lacks objective and quantitative parameters. This study compared the acute effects of different KT interventions on the local thresholds of pressure pain and current perception in healthy adults to determine the potential mechanisms by which KT relieves pain.

**Methods:**

Thirty healthy female subjects randomly received four KT interventions, namely, no taping (NT), placebo taping (PT), Y strips of KT (KY), and fan strips of KT (KF), on the waist. Current perception threshold (CPT), pressure pain threshold (PPT), soft tissue hardness, and the visual analog scale (VAS) scores of the subjects’ perceived pain were immediately measured after taping. Repeated-measures ANOVA was performed to determine significant differences in these parameters among the four interventions.

**Results:**

Significant differences in CPT values among the interventions were observed at the frequency of 5 Hz (*F* = 3.499, *p* = 0.019, η_*p*_^2^ = 0.111). *Post hoc* analysis revealed that CPT was significantly higher for KF than for NT (*p* = 0.008, 95% CI = 1.390–11.990). Significant differences in PPT values (*F* = 4.352, *p* = 0.012, η_*p*_^2^ = 0.130) and soft tissue hardness (*F* = 2.957, *p* = 0.049, η_*p*_^2^ = 0.093) were observed among the different taping conditions. *Post hoc* analysis revealed that PPT was significantly higher for KF than for PT (*p* = 0.011, 95% CI = 0.071–0.749), and soft tissue hardness was significantly higher for KF than for NT (*p* = 0.010, 95% CI = 0.461–4.586) and KY (*p* = 0.040, 95% CI = 0.059–3.800). No significant differences in self-perceived pain among the interventions were observed.

**Conclusion:**

The healthy adult females had higher PPT values, lower soft tissue hardness, and higher CPT values at 5 Hz under KF intervention applied on the waist than those under the other taping interventions. Moreover, the different taping conditions had no significant differences in terms of VAS of perceived pain. These results provide guidance for the application of KT on pain management.

## Introduction

Kinesio taping (KT) was invented by Kenso Kase in the 1970s. It is a thin and ventilated waterproof elastic tape that can stretch by as much as 120%–140% of its initial length. KT is a relatively new method that has become a simple and effective treatment for musculoskeletal diseases (Kase et al., 2003; Parreira Pdo et al., 2014; Hosp et al., 2018). According to the characteristics of the target human muscle and joint shape, KT is cut into I, Y, X, O, or fan strips or other shapes as necessary. I and Y strips are commonly used for functional correction, whereas fan strips are typically utilized to increase sensory input, relieve pain, and reduce swelling (Kumbrink, 2014; Banerjee et al., 2017; Chen and Yu, 2017; Vercelli et al., 2017).

To date, the effects of KT interventions on pain have not been verified yet. Previous studies reported that KT has good efficacy in relieving shoulder pain, knee pain, Achilles tendon pain, and chronic lower back pain (Mostafavifar et al., 2012; Kalron and Bar-Sela, 2013). Several systematic reviews that explored the effects of KT on patients with musculoskeletal diseases reported that KT can reduce pain intensity, especially in the short term (Mostafavifar et al., 2012; Kalron and Bar-Sela, 2013; Morris et al., 2013). Recent studies proposed that the possible mechanism by which KT relieves pain proceeds as follows. First, after KT is applied, the skin is lifted by folds, the space between superficial skin and its associated underlying connective tissues increases, blood and lymph circulation accelerates, and inflammatory factors are dissipated, thereby reducing pain (Kase et al., 2003). Subsequently, mechanical stimulation of the skin through KT increases the afferent stimulation of large-diameter nerve fibers and reduces the afferent input received by small-diameter nerve fibers, such as nociceptors, thereby reducing pain sensation (i.e., gate theory) (Thelen et al., 2008; Pamuk and Yucesoy, 2015). Afterward, KT increases blood circulation and muscle temperature by stimulating the vasomotor reflex, and the associated increase in metabolic activity may reduce pain (Kirmizigil et al., 2019; Liu et al., 2020). Finally, owing to the sense of stability and security generated by KT, the psychologically expected response of the subjects may increase, thereby inducing placebo effects (Mak et al., 2019; Yin and Wang, 2020). However, this series of processes is only a hypothesis deduced from experimentally observed phenomena. Moreover, the current methods for assessing pain are merely functional scales or visual analog scores (VAS) (Parreira Pdo et al., 2014; Boobphachart et al., 2017; Kakar et al., 2019; Kirmizigil et al., 2019; Rahlf et al., 2019). Experimental results reported in the literature are subjective, variable, and uncertain and thus cannot provide an objective and quantitative standard for pain assessment.

Other researchers presented unfavorable assessments about KT. Parreira Pdo et al. (2014) reported that KT failed to substantially improve the pain symptoms of patients with chronic low back pain. Kalichman et al. (2016) also showed that KT did not improve the shoulder pain symptoms of patients with hemiplegia in the short term. Only a few high-quality studies of KT have been conducted. The existing literature cannot provide a clear guidance for the clinical applications of KT.

Current perception threshold (CPT) is an indicator that can be used to test non-invasive quantitative sensory nerve fiber functions, as well as to determine the minimum current that triggers and intensifies pain stimulus. In addition, a CPT detector automatically converts the output CPT values (Rendell et al., 1989). This device can be used to conveniently, rapidly, and non-invasively measure CPT in a single or several parts of the body. CPT detectors are widely used in clinical quantitative evaluation of sensitivity (Baquis et al., 1999; Yin et al., 2018). Through a pair of gold-plated electrodes, the detector sends three types of harmless sinusoidal alternating current stimulations at different frequencies (5, 250, and 2000 Hz) to the body (Baquis et al., 1999; Yao et al., 2004). In turn, these frequencies can selectively stimulate different subgroups of sensory nerve fibers: 5, 250, and 2000 Hz mainly stimulate unmyelinated C nerve fibers, fine myelinated Aδ nerve fibers, and coarse myelinated Aβ nerve fibers, respectively (Kenshalo et al., 1968; Koga et al., 2005; Félix et al., 2009), at current stimulation intensities ranging from 0.01 mA to 9.99 mA (Katims et al., 1987). The current intensity of each stimulation in the test process of CPT detectors is constant with good repeatability and limited interference from various factors, such as skin mucosal thickness, humidity, temperature, scar or edema (Yin et al., 2018).

The present study aimed to explore the effects of different KT interventions on the local thresholds of current and pain perception in healthy adults. Moreover, this study preliminarily explored the influence of different KT strips or shapes on pain. The results provide a more scientific and reliable guidance for KT applications in clinical settings. We hypothesized that fan strips of KT (KF) will substantially increase CPT values.

## Materials and Methods

### Participants

A minimum of 21 participants were required from a power of 0.95, an effect size of 0.25, a significance level of 0.05 in repeated-measures ANOVA, then considering a 15% drop rate, 30 female participants from a local university were recruited on the basis of the following inclusion criteria: healthy female college students, with no sensory disturbance, with prior knowledge of KT, and with a BMI of 18.5 ≤ BMI ≤ 23.9. Participants were excluded if they had a history of alcohol consumption, cervical and lumbar diseases, central nervous system diseases, diabetes, cancer, implanted electronic devices (such as pacemakers), wound(s) at the site of measurement, menstruating during the study period, allergic to KT and refused to provide a signed informed consent. The baseline characteristics of the participants are summarized in Table 1.

**TABLE 1:** Participants’ characteristics ($\bar{x}$ ± *s*).

N	Sex	Age/years	Height/cm	Weight/kg	BMI/kgm^–2^
30	Female	21.0 ± 1.2	161.2 ± 5.6	54.6 ± 7.0	21.1 ± 3.1

All participants were instructed to read, understand and sign the informed consent form. This study was approved by the Ethics Committee of the Shanghai University of Sport (No. 102772020RT038).

### Experimental Protocol

#### Preparation

Prior to the test, the tester described the specific procedures of the experiment to the participants. The tester explained what the test would contribute to the study and emphasized its potential risks and benefits. Moreover, the tester clarified the purpose and practical significance of the study. The participants were given an informed consent form for exhaustive reading. After they explicitly stated that they clearly understood the experimental content, they were then asked to sign the informed consent form. During the test, indoor temperature was adjusted to 24.5 ± 0.5°C and humidity was maintained at 40%.

#### Taping Interventions

**FIGURE 1:**
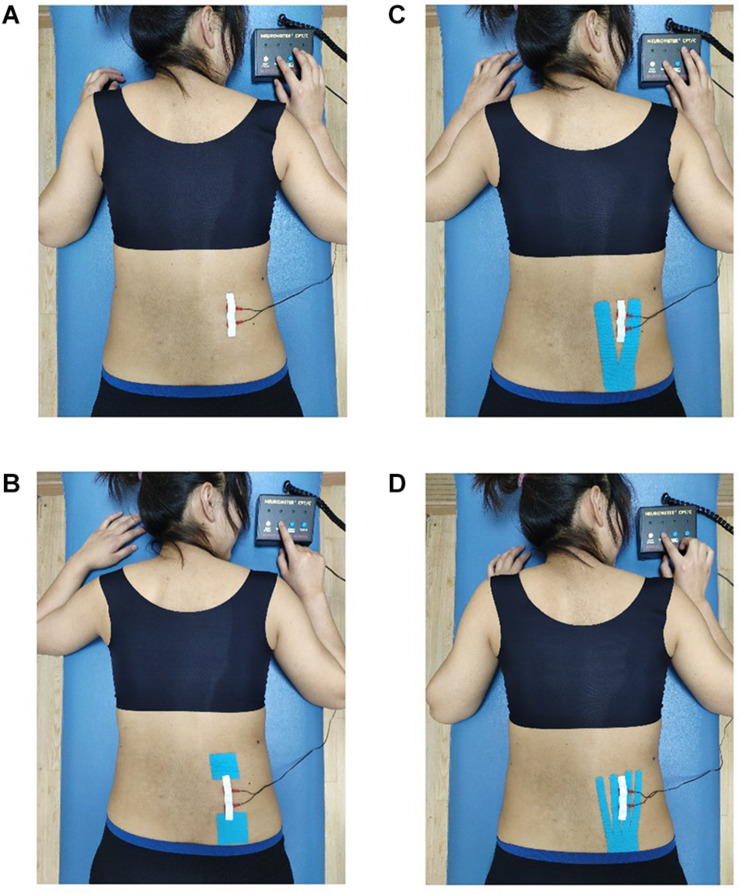
Different taping methods with the CPT test. **(A)** No taping, **(B)** placebo taping, **(C)** Y-strip of Kinesio taping, and **(D)** fan-strip of Kinesio taping.

Kinesio Tex Gold (5 cm × 5 m, United States; Matheus et al., 2017) kinesiology tapes were used in this study. Prior to taping, the area was shaved and wiped clean with alcohol. Each participant received four different KT interventions, namely, no taping (NT), placebo taping (PT), Y strip of KT (KY) and fan strip of KT (KF) (Figure 1). The tape was applied by a KT-certified therapist who was blinded to the purpose of the experiment. The participants were deliberately not informed about the different interventions effects of taping. The order of taping was counterbalanced and randomized. A 1-week washout phase was assigned between each taping intervention to limit any learning effect. For example, if the participant received the first random taping intervention on a Monday, then the second random taping condition would be performed also on Monday the following week.

According to the proportional distribution law of KT tension, Y strips were equally divided into two tails, whereas fan strips were evenly split into four tails (Yu et al., 2016). For NT, the participants were not taped with KT. For PT, a pair of 5 cm × 5 cm tension-free tapes was applied at both ends of the measured site. While the tapes were applied, the participants were asked to stand in an anatomical position, that is, bent forward, legs kept straight, their middle finger touching the back of their feet to extend their lower back, the posterior superior iliac spine was used as the location to anchor the tape and KT was extended up to the right L1–L5 erector spinal regions (Liu et al., 2020). Following the KT user manual, the tension of the tapes was set to approximately 10%–15% (Figure 1; Kase et al., 2003).

#### CPT Test

The CPT test was conducted using a Neurometer^®^ CPT/C Quantitative sensory nerve detector (Neurotron Inc., Baltimore, MD, United States) via a standardized automatic double-blind test method. First, the skin was scrubbed and cleaned. Subsequently, a pair of alloy electrodes (1 cm diameter, 1.7 cm distance) were then coated with a layer of hypoallergenic conductive electrode gel and then fixed on the region of interest (ROI) with an adhesive plaster.

Prior to the actual test, an automatic strength calibration was performed to automatically determine the range of current intensity for each participant for the subsequent automatic cycle test. After the electrode was secured, the participant was instructed to press the “test” button of the remote control. The intensity of electrical stimulation would gradually increase. The participant was instructed to release the button once electrical stimulation was felt, and the calibration was repeated several times until the range of current intensity was established.

Using the remote control, the participants were tested at 5, 250, and 2000 Hz. As soon as the participants pressed the “test” button, the device would output “stimulus A–rest–stimulus B” in response. The participants were then instructed to judge the strength of both stimuli. They were asked to press the button corresponding to the stimulus whose intensity they thought was higher. If they did not feel any stimulation or could not tell the difference in intensity between both stimuli, they were required to press the “rest” button. This cycle was repeated several times, and the CPT device automatically adjusted the intensity of the next stimulus on the basis of the participants’ feedback. The detector automatically provided the threshold of current perception according to the test results, and the total test time of each subject did not exceed 15 min (Griffioen et al., 2018).

#### PPT and Hardness Tests

PPT and hardness values were determined using a tissue hardness algometer (OE-220 Tissue Hardness–Algometer Combo, Japan). The application surface (a 1 cm^2^ round rubber tip and a disk) was placed in the test position. For the PPT test, the tester slowly and evenly increased the pressure vertically downward, and the participants were asked to press the remote button as soon as they felt pain. For the hardness test, the tester slowly and evenly increased the pressure vertically downward until the digital display automatically outputted the value. The two tests were repeated five times, and average values were calculated as the PPT and hardness values.

#### Selection of ROI

The ROI, located at the site of the erector spinae that was not covered by the tapes, was vertically placed 5–6 cm away from the axis of the spine and parallel to the axis (Liu et al., 2020). Y strips were cut into two equal tails 2.5 cm in width each (tape width was 5 cm), whereas fan strips were cut into four equal tails 1.25 cm in width each to ensure that the ROI of each taping condition was consistent. The end point of the tail closest to the spine was about 1.5 cm vertically away from the spine, and the tails were taped in equal distribution. In this way, the width of the exposed skin area between the end points of each tail of the Y strips was about 3 cm, whereas the width of the exposed skin area between the end points of each tail of the fan strips was about 1 cm. Thus, the ROI of the exposed skin area between the 2nd and 3rd end points of the fan strips coincided with the ROI of the exposed skin area between the two end points of the Y strips; the ROI of both strips was about 5–6 cm away from the spine axis (Figure 1).

#### VAS Test

The electrical stimulation generated by CPT during the test would cause slight, harmless and uncomfortable pain, this study investigated if KT has inhibitory effect for pain. Therefore, the participants were instructed to describe the degree of self-perceived pain in their waist after applying different KT interventions. They were then asked to provide VAS scores of increasing pain perception ranging from 0 to indicate “without pain” to 10 to denote “with great pain.”

### Statistical Analysis

The quantitative data followed a normal distribution and were presented as mean ± standard deviation ($\bar{x}$ ± s). One-way repeated-measures ANOVA was used to determine significant differences among the parameters of NT, PT, KY, and KF interventions. Bonferroni test was used for *post hoc* analysis, and the significance level was set at *p* < 0.05. Moreover, 95% confidence interval (CI) was determined, and effect size was expressed as η_*p*_^2^. Effect size was considered small, moderate or large if 0.01 ≤ η_*p*_^2^ < 0.06, 0.06 ≤ η_*p*_^2^ < 0.14, or η_*p*_^2^ ≥ 0.14, respectively. All data were analyzed using IBM SPSS software version 19.0 (Chicago, IL, United States).

## Results

**FIGURE 2:**
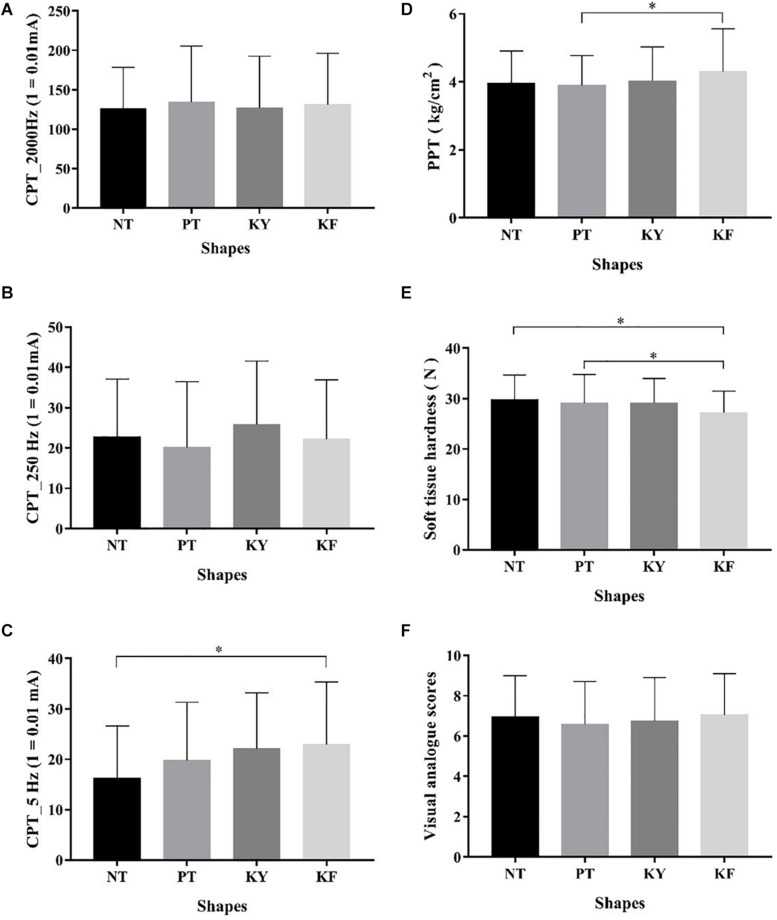
Comparison of different taping methods in terms of CPT, PPT, Hardness, and VAS scores ($\bar{x}$ ± *s*). **(A)** Different taping methods in terms of CPT with 2000 Hz; **(B)** different taping methods in terms of CPT with 250 Hz; **(C)** different taping methods in terms of CPT with 5 Hz; **(D)** different taping methods in terms of PPT; **(E)** different taping methods in terms of soft tissue hardness; and **(F)** different taping methods in terms of visual analogue scores. *Significant difference between groups, *p* < 0.05.

No significant differences in CPT values were found among the different interventions at 2000 Hz (*p* = 0.912, *F* = 0.006, η_*p*_^2^ = 0.147) and 250 Hz (*p* = 0.476, *F* = 0.839, η_*p*_^2^ = 0.028) (Figure 2). By contrast, significant differences were observed in acute effects among the different taping methods at 5 Hz (*p* = 0.019, *F* = 3.499, η_*p*_^2^ = 0.111). *Post hoc* test revealed that KF had significantly higher CPT values than NT (*p* = 0.008, 95% CI = 1.390–11.990) (Figure 2 and Table 2).

**TABLE 2:** Comparison of the different taping methods in terms of CPT ($\bar{x}$ ± *s*; 1 CPT = 0.01 mA).

Frequency	NT	PT	KY	KF	*F*-value	*p-*value	η*_*p*_^2^*
2000 Hz	126.17 ± 52.22	134.20 ± 71.39	127.23 ± 65.36	131.13 ± 65.20	0.177	0.912	0.006
250 Hz	22.75 ± 14.35	20.12 ± 16.33	25.87 ± 15.69	22.20 ± 14.70	0.839	0.476	0.028
5 Hz	16.24 ± 10.39	19.83 ± 11.47	22.10 ± 11.09	22.93 ± 12.40^*a*^	3.499	0.019*	0.111

*NT, no taping; PT, placebo taping; KY, Y-strip of Kinesio taping; and KF, fan-strip of Kinesio taping. *F*, statistic value of variance analysis, and η*_*p*_^2^*, effect size. ^*a*^ Significant difference between KF and NT, *p* < 0.05.^∗^Significant difference between groups, *p* < 0.05.*

Significant differences in acute effects on PPT values were observed among the different interventions (*p* = 0.012, *F* = 4.352, η_*p*_^2^ = 0.130). *Post hoc* test revealed that KF had significantly higher PPT values than PT (*p* = 0.011, 95% CI = 0.071–0.749) (Figure 2 and Table 3).

**TABLE 3:** Comparison of the different taping methods in terms of PPT (kg/cm2), Hardness (N), and VAS scores ($\bar{x}$ ± *s*).

	NT	PT	KY	KF	*F-*value	*p-*value	η*_*p*_^2^*
PPT	3.96 ± 0.94	3.90 ± 0.88	4.03 ± 1.00	4.31 ± 1.26^a^	4.352	0.012*	0.130
Hardness	29.73 ± 4.94	29.11 ± 5.68	29.14 ± 4.86	27.21 ± 4.28^*b*^	2.957	0.049*	0.093
VAS	6.96 ± 2.03	6.59 ± 2.13	6.75 ± 2.15	7.05 ± 2.05	1.646	0.185	0.054

NT, no taping; PT, placebo taping; KY, Y-strip of Kinesio taping; and KF, fan-strip of Kinesio taping. PPT, pressure pain threshold; Hardness, soft tissue hardness; and VAS, visual analog scores. *F*, statistic value of variance analysis, and η*_*p*_^2^*, effect size. ^*a*^Significant difference between KF and PT, *p* < 0.05. ^*b*^ Significant difference between KF and NT, KF and KY. *Significant difference between groups, *p* < 0.05.

Significant differences in acute effects on hardness values of soft tissues were observed among the different interventions (*p* = 0.049, *F* = 2.957, η_*p*_^2^ = 0.093). *Post hoc* test revealed that KF had significantly higher hardness values than NT (*p* = 0.010, 95% CI = 0.461–4.586) and KY (*p* = 0.040, 95% CI = 0.059–3.800) (Figure 2 and Table 3).

No significant difference in pain VAS scores were observed among the different interventions (*p* = 0.185, *F* = 1.646, η_*p*_^2^ = 0.054) (Figure 2 and Table 3).

## Discussion

### Effects of KT on Pain Perception

Results showed that KT intervention had a certain effect on CPT and PPT values. Moreover, KT intervention at 5 Hz had the most significant effect on the participants, indicating that this type of intervention had a positive effect on, and was the most sensitive to, pain perception. PPT is widely used in clinical practice as a semiobjective method for quantifying targeted pain (Fischer, 1987; Persson et al., 2004). Results also showed that KF intervention had a significantly high effect on PPT values, consistent with the results of previous studies that reported that KT intervention can evidently improve the pressure in patients with acute and chronic disease pain thresholds (González-Iglesias et al., 2009; Kaya et al., 2011), thereby relieving pain. Chang et al. (2012) stated that the PPT values of baseball players with medial epicondylitis of the forearm and healthy people remarkably increase after KT intervention. They argued that this increase in PPT values is related to the reduction of muscle and fascia tension. In previous studies, KT intervention exhibited good clinical efficacy in alleviating common musculoskeletal disorders, such as delayed muscle pain, knee osteoarthritis pain, low back pain in pregnancy and mechanical neck pain (Saavedra-Hernández et al., 2012; Kaplan et al., 2016; Kakar et al., 2019; Kirmizigil et al., 2019). The present study further suggested that this clinical efficacy is possibly related to the interference of KF intervention to the input of sensory information to C nerve fibers, resulting in the thin non-myelinated nerve fibers in the human skin to exhibit a dull response to pain, thereby inducing nociceptive pain relief. In theory, PPT can be used as a reference for the clinical applications of KT intervention.

### Effects of KF Intervention on Pain Suppression

Changes in incoming inputs allegedly produce inhibitory effects by blocking nociceptive inputs, thereby reducing pain in the short term (Bockrath et al., 1993; Aytar et al., 2011). Banerjee et al. (2017) observed that the pain in the anterior thoracic region of patients with cancer is considerably reduced when KF tapes are anchored near their axillary lymph nodes and attached to their ribs, diaphragm and upper abdomen. In an earlier case report, Banerjee et al. (2016) reported that the addition of a motion-mechanics tape to soft tissue therapy of patients with secondary breast cancer remarkably reduced musculoskeletal pain by approximately 50% and “tearing” and “burning” sensations by about 85% compared with pretreatment. According to the gate control theory, stimulating low threshold skin mechatronic receptors inhibit nociceptive fibers to reduce pain in the relevant dermis (Bockrath et al., 1993; Thelen et al., 2008; Aytar et al., 2011).

In the present study, the effects of KY intervention on pain sensitivity were not significant, suggesting that the effects are related to tape shapes. KT intervention can reportedly cause measurable muscle deformation at the site of tape application and adjacent tissues (Pamuk and Yucesoy, 2015). Moreover, this intervention can allegedly rapidly decrease shear stiffness generated by the shallow depth of the muscles at the KT application site (Wang et al., 2019). Other studies stated that skin deformation after KT intervention may stimulate neuromuscular pathways (Paoloni et al., 2011; Álvarez-Álvarez et al., 2014). This deformation may be related to the temporary pressure gradient in the tissues (Pamuk and Yucesoy, 2015). Taping formed small skin folds in the participant’s waist because KT is viscoelastic. KF intervention had more tail branches and a wider coverage than KY intervention. Results showed that the size of skin folds formed by KF intervention was considerably larger than that of KY intervention, thereby forming a large pressure gradient difference. In turn, this gradient difference might have produced a wider range of deformation and mechanical sensor stimulation on the skin. Kakar et al. (2019) speculated that tape length and coverage area rather than the type of taping are the key to effectively reduce pain. Compared with KY intervention, KT intervention may be more effective in reducing pain because it can cover a larger area and possibly encompass the entire associated dermoid around the knee joint. Results suggested that KF intervention would interfere with the input of C nerve fibers, thereby reducing pain sensation. Hence, KF intervention may slightly decrease or dull the pain perception of human skin. In general, the CPT values after KT intervention increased, indicating that the intervention obstructed nerve conduction velocity. By comparison, the CPT values increased most significantly after KF intervention. These results indicated that the KT intervention had a greater influence on human nerve conduction than KF intervention because the former had a higher bifurcation and coverage.

Through musculoskeletal ultrasonic measurements, Yu et al. (2016) confirmed that the natural tension caused as KT tapes are attached to the skin increases the subcutaneous space by about 0.2 mm. In subcutaneous tissues, the blood capillary plexus is about 0.3–0.7 mm below the skin surface. Taping extends the formation of capillary network upward to the skin below about 0.04–0.08 mm (Swain and Grant, 1989). Thus, KT intervention can change the spatial structure and arrangement of the position of the shallow capillary network. Liu et al. (2020) reported that compared with other interventions, KF intervention substantially increases skin temperature after 10 min, thereby promoting blood and lymph flow, which in turn dissipates inflammatory factors to alleviate pain (Zainuddin et al., 2005). However, this conclusion must be verified experimentally and clinically.

Several studies claimed that the pain caused by blunt stress stimulation is related to C fiber (Beissner et al., 2010). The participants in the present study had the highest CPT values when stimulated at 5 Hz because small myelinated C fibers are most sensitive to this frequency. Stimulation of C nerve fibers at 5 Hz mainly reflects various forms of nociception. Although the relationship between CPT and PPT values could not be directly determined in this study, this result nonetheless provides an important reference for verifying the role of KT intervention in the acute effects of pain.

### Effects of KT Intervention on Soft Tissue Hardness

Soft tissue hardness is defined as the resistance of soft tissues to vertical pressure (Murayama et al., 2000, 2005). Changes in soft tissue hardness may affect the pain perception of the human skin. Weerapong et al. (2005) reported that changes in muscle flexibility, rearrangements of muscle structure and relaxation of muscle fibers help in relieving pain. They argued that these muscular modifications block pain signals through presynaptic inhibition or increase PPT values by reducing or preventing pain signals from reaching the level of consciousness. Few studies explored the influence of KT intervention on soft tissue hardness. Kim and Lee (2018) noted that the hardness of the upper trapezius muscle remarkably decreased and the PPT value substantially increased after applying the sternocleidomastoid muscle relaxation technique. Via magnetic resonance elastography, Wang et al. (2019) observed the acute effects of KT intervention on a single side of the lumbar paraspinal muscle and found that the intervention reduced muscle stiffness near the taping area. By pulling more space between the skin and the subcutaneous fascia, KT improves blood and lymph flow, thereby reducing muscle stiffness. Pajero Otero et al. (2019) applied KF intervention to treat breast cancer-related lymphedema. They found that KT intervention has a greater curative effect and lower soft tissue hardness than pressure clothes. However, they evaluated the effects of these interventions only on patients with edema. Moreover, they obtained these results after a long period of treatment. But, our results merely showed the acute effects of these interventions on healthy people, and failed to establish the direct cause of these effects.

The present study showed that KF intervention rendered the skin more flexible after lifting up the skin folds formed by strong mechanical stimulations to the skin. According to Weerapong et al. (2005), skin flexibility decreases skin hardness and reduces the pain of presynaptic inhibition. By contrast, NT and PT interventions did not produce skin deformations and wrinkles, and thus soft tissue hardness after these interventions did not change.

### Effects of KT Intervention on VAS Scores

Rahlf et al. (2019) reported that KT intervention considerably reduces pain VAS scores. By contrast, the present study did not find significant differences in VAS scores among the four interventions likely because of the fact that this was a blinded study to exclude psychological expectations of the participants. During the experiment, the participants were asked to assume a static prone position, and they were not informed about the intervention that would be applied in each experiment. Moreover, the material and thickness of KT used in this study was similar to the human skin. Therefore, the participants probably did not feel the tapes, or if they did, they could not tell the difference between the different interventions. These conditions probably minimized psychological expectations and placebo effects to a certain extent.

### Limitations

This study has several limitations that must be considered when interpreting the results. The sample size was small. Only the acute effects of KT intervention on the participants under static conditions in the short term were evaluated, and therefore, the effect of KT under dynamic conditions remains unclear. Moreover, this study included healthy female subjects only. Thus, the results may not be applicable under pathological conditions or in male subjects. Furthermore, placebo taping should fulfill the conditions for both experimental situations while maintaining the format and place of application to expand the applicability of the results, and other materials aside from Kinesio tapes may be used. Finally, the test site was limited to the waist, and thus the result may not completely apply to other joints or parts of the body.

### Future Research

Researchers should aim to conduct high-quality and blinded randomized controlled trials on the effects of KT interventions on pain relief. The number of male subjects should be increased, and future studies should include volunteers with various pathologies. The time for observing the effects of various interventions should be extended. Other types of spatial supports, including drift taping and other taping methods, may be used. The relationship between the application times and effects of different taping interventions on improving local skin pain inhibition must be quantitatively analyzed in combination with physiological changes in cells and blood.

## Conclusion

Compared with that of the other interventions, KF intervention produced higher PPT values in the waist skin of healthy female subjects. Moreover, this intervention resulted in lower soft tissue hardness and higher CPT values at 5 Hz. No significant differences in the VAS scores of perceived pain were observed among the different interventions. The results provide guidance for the application of KT in pain management. Nevertheless, further research exploring the mechanism by which KT relives pain is warranted.

## Data Availability Statement

The raw data supporting the conclusions of this article will be made available by the authors, without undue reservation.

## Ethics Statement

The studies involving human participants were reviewed and approved by the Ethics Committee of the Shanghai University of Sport (no. 102772020RT038). The patients/participants provided their written informed consent to participate in this study.

## Author Contributions

KL contributed to the subject recruitment, data collection, and manuscript writing. LY contributed to the subject recruitment and data collection. ZM and BY undertook the statistical analysis. YM and LH conceived the study and interpreted the results. All authors contributed to the article and approved the submitted version.

## Conflict of Interest

The authors declare that the research was conducted in the absence of any commercial or financial relationships that could be construed as a potential conflict of interest.
